# Effects of long-term balance training with vibrotactile sensory augmentation among community-dwelling healthy older adults: a randomized preliminary study

**DOI:** 10.1186/s12984-017-0339-6

**Published:** 2018-01-18

**Authors:** Tian Bao, Wendy J. Carender, Catherine Kinnaird, Vincent J. Barone, Geeta Peethambaran, Susan L. Whitney, Mohammed Kabeto, Rachael D. Seidler, Kathleen H. Sienko

**Affiliations:** 1 Department of Mechanical Engineering, University of Michigan, 2350 Hayward St., Ann Arbor, 48109 MI USA; 2 Department of Otolaryngology, Michigan Medicine, University of Michigan, 1500 E Medical Center Dr, Ann Arbor, MI 48109 USA; 3 Physical Medicine & Rehabilitation, Michigan Medicine, University of Michigan, 1500 E Medical Center Dr, Ann Arbor, MI 48109 USA; 4 Department of Physical Therapy and Otolaryngology, School of Health and Rehabilitation Sciences, University of Pittsburgh, 4028 Forbes Tower, Pittsburgh, PA 15260 USA; 5 Department of Internal Medicine, University of Michigan, 1500 East Medical Center Drive, Ann Arbor, 48109 MI USA; 6 School of Kinesiology, University of Michigan, 1402 Washington Heights, Ann Arbor, MI 48109 USA; 7 Department of Psychology, University of Michigan, 530 Church St, Ann Arbor, MI 48109 USA; 8 Department of Applied Physiology and Kinesiology, University of Florida, Gainesville, USA; 9 Department of Biomedical Engineering, University of Michigan, 2200 Bonisteel Blvd, Ann Arbor, MI 48109 USA

**Keywords:** Balance rehabilitation, Vibrotactile, Biofeedback, Sensory augmentation, Long-term, Older adults, Home-based, Smartphone balance trainer, Telerehabilitation, Wearable devices

## Abstract

**Background:**

Sensory augmentation has been shown to improve postural stability during real-time balance applications. Limited long-term controlled studies have examined retention of balance improvements in healthy older adults after training with sensory augmentation has ceased. This pilot study aimed to assess the efficacy of long-term balance training with and without sensory augmentation among community-dwelling healthy older adults.

**Methods:**

Twelve participants (four males, eight females; 75.6 ± 4.9 yrs) were randomly assigned to the experimental group (*n* = 6) or control group (n = 6). Participants trained in their homes for eight weeks, completing three 45-min exercise sessions per week using smart phone balance trainers that provided written, graphic, and video guidance, and monitored trunk sway. During each session, participants performed six repetitions of six exercises selected from five categories (static standing, compliant surface standing, weight shifting, modified center of gravity, and gait). The experimental group received vibrotactile sensory augmentation for four of the six repetitions per exercise via the smart phone balance trainers, while the control group performed exercises without sensory augmentation. The smart phone balance trainers sent exercise performance data to a physical therapist, who recommended exercises on a weekly basis. Balance performance was assessed using a battery of clinical balance tests (Activity Balance Confidence Scale, Sensory Organization Test, Mini Balance Evaluation Systems Test, Five Times Sit to Stand Test, Four Square Step Test, Functional Reach Test, Gait Speed Test, Timed Up and Go, and Timed Up and Go with Cognitive Task) before training, after four weeks of training, and after eight weeks of training.

**Results:**

Participants in the experimental group were able to use vibrotactile sensory augmentation independently in their homes. After training, the experimental group had significantly greater improvements in Sensory Organization Test and Mini Balance Evaluation Systems Test scores than the control group. Significant improvement was also observed for Five Times Sit to Stand Test duration within the experimental group, but not in the control group. No significant improvements between the two groups were observed in the remaining clinical outcome measures.

**Conclusion:**

The findings of this study support the use of sensory augmentation devices by community-dwelling healthy older adults as balance rehabilitation tools, and indicate feasibility of telerehabilitation therapy with reduced input from clinicians.

## Background

Age-related deterioration of sensory function, inefficient integration of sensory systems, and reduced muscle strength contribute to decreased balance performance in older adults [1–3]. Degradation of balance performance increases fall risk [4–6] and fear of falling [7], and inhibits mobility, thereby reducing independence and quality of life [8, 9].

Exercise-based rehabilitation programs are effective for improving balance performance in community-dwelling older adults [10]. Typical regimens emphasize building strength and stamina to counteract musculoskeletal degeneration, and balance training to isolate and challenge the somatosensory, visual, and vestibular systems [10, 11]. In particular, balance training leverages the ability of the central nervous system to “reweight” functioning sensory inputs [12]. Clinic-, group-, and home-based balance training programs guided by clinicians and lasting four to twelve weeks have yielded significant improvements in clinical outcome measures (e.g., Sensory Organization Test, Timed Up-and-Go) corresponding directly to reductions in fall rate, occurrence, and risk, and maintenance of social and physical activity [13–16]. Training programs without direct clinical guidance, however, are less effective [17, 18]. Kao et al. reported that more participants in a supervised exercise program group showed clinically significant improvements than those performing the same exercises at home without supervision [17]. Lacroix et al. showed that a 12-week supervised balance and strength training program improved static, dynamic, proactive, and reactive measures of balance more than an unsupervised program in healthy older adults [18]. These studies suggest that monitoring performance and providing feedback during balance and strength training may improve program efficacy.

Sensory augmentation (SA) provides additional information to complement and/or replace native sensory input from the somatosensory, visual, and/or vestibular systems [19]. SA systems for balance applications typically employ one sensor or a network of sensors (e.g., motion capture, force plate, inertial measurement unit, goniometer) to measure body motion, and a display to communicate body motion and/or provide instructional cues. Most studies of balance-related SA have explored real-time usage applications as an assistive device in lieu of the somatosensory contact cues provided by a cane or walker to the fingertips [19–34]. During real-time use, it is hypothesized that the central nervous system incorporates SA as an additive input, supplementing other sensory information [22, 35, 36]. In individuals with balance deficits, healthy young adults, and healthy older adults, various SA modalities (e.g., vibrotactile [23–29], visual [30], auditory [31, 32], electrotactile [19, 37], and multi-modal [33]) have been shown to improve real-time balance performance by reducing body sway during static, dynamic, and perturbed standing tasks. While use of SA based on trunk sway during gait tasks (e.g., tandem walk, straight walk) has been shown to reduce trunk sway, the effects are limited [32, 38–40].

In addition to real-time assistive device applications, SA may be useful as a rehabilitation tool during traditional balance training. It is hypothesized that cues from SA may facilitate the central nervous system in “reweighting” sensory inputs during training to improve balance ability [22, 41]. To evaluate whether SA devices can be used as rehabilitation tools, it is important to analyze whether balance improvements observed during training persist after training is completed and SA is no longer provided.

Prior studies have shown that post-training improvements persist hours to days following short-term (i.e., less than one week) training with SA [23, 33, 42] and on the order of weeks to months following multi-session (i.e., more than one week) training with SA for people with balance deficits [43–45]. Rossi-Izquierdo et al. showed that after two weeks of exercise training with vibrotactile SA, people with Parkinson’s disease reduced trunk sway on trained exercises and demonstrated improved clinical outcome measures (e.g., Dizziness Handicap Inventory, Sensory Organization Test) [43]. Furthermore, these improvements persisted three months after training [43]. Basta et al. reported similar improvements in a group of people with various balance disorders trained with vibrotactile SA, but found no such effect in a group trained with erroneous SA signals [44]. Brugnera et al. found improved clinical balance measures (e.g., Sensory Organization Test, Activities-specific Balance Confidence) among people with vestibular disorders following two weeks of balance training with vibrotactile SA, but found no improvements among participants trained following standard rehabilitation practices [45].

Limited studies have examined balance improvements after longer-term training with SA among healthy older adults. Video game–based in-home balance training was shown to improve clinical measures (e.g., maximal muscle strength, Activity-specific Balance Confidence, risk of falling) after a minimum of five weeks of training [46–49]. However, video game–based balance training typically requires a balance platform (e.g., Wii Fit balance board) and a display screen to provide visual cues, which can limit its utility during balance exercises that require closed eyes, head movements, and altered stances. Lim et al. used multi-modal SA to investigate balance improvements after a two-week balance training program involving 36 healthy older adults [50]. All participants wore a SA device (SwayStar™), but only the experimental group received SA. Participants’ trunk sway was monitored as they trained on the same seven standing and gait tasks for two consecutive weeks (3×/week). Both experimental and control groups showed reduced body sway during the final training session, but training with SA provided little benefit over training alone. For most tasks, sway reductions did not persist in either group during immediate and one-month post-training assessments. However, given that balance training is most effective following longer training periods (i.e., up to 12 weeks [16]), balance improvements and the persistence of the potential improvements between the groups may not have been realized given the relatively short training period.

To understand the efficacy of SA as a rehabilitation tool among community-dwelling older adults, this preliminary study investigated balance improvements after long-term (eight weeks) balance training with and without SA. We hypothesized that all participants would show improved clinical outcome scores after training, but that participants receiving SA would show greater improvements.

## Methods

### Participants

Twelve community-dwelling healthy older adults were recruited to participate following a screening session. The sample size was partially informed by single day SA study findings [23, 33, 51]. Participants in the greater Ann Arbor, MI area were recruited via flyers and online advertisements on the website umhealthresearch.org. The recruiting period started in 2014 and ended in 2016. Participants were eligible for inclusion if they were 65–85 years of age; medically stable; scored more than 26 points on the Montreal Cognitive Assessment; could stand unassisted for ten minutes; reported balance concerns (≥1 confirmative answer to balance perception questions, e.g., fear of falling, falls in the past year, losses of balance in the past 12 months, balance ratings ≥2 on a five-point scale, Fig. 2); and could walk the distance of a city block without using an assistive device. Participants who had sustained a fall that required hospitalization or serious injury, had severe uncorrected vision or hearing loss, had a lower extremity fracture or sprain in the last six months or previous lower extremity joint replacement, had a history of a neurological condition (e.g., Parkinson’s disease, multiple sclerosis, stroke), had motion-provoked vertigo or a diagnosed vestibular deficit, or had a body mass index larger than 30 kg/m^2^ were excluded.

The twelve participants were randomly assigned to the experimental group (EG) or control group (CG) before pre-training assessments with a one-to-one allocation ratio. The EG (*n* = 6, 76.2 ± 5.5 yrs., 1 male/5 females) received vibrotactile SA during the training, while the CG completed the training without vibrotactile SA (*n* = 6, 75.0 ± 4.7 yrs., 3 males/3 females). The study team randomized the participant assignments by blindly drawing sealed slips of paper with group designations. The first two participants were randomized in one block and the following ten participants were randomized in a second block. All participants gave written informed consent and the study was conducted in accordance with the Declaration of Helsinki. The study was reviewed and approved by the University of Michigan Institutional Review Board (HUM00086479).

### Protocol

**Fig. 1:**
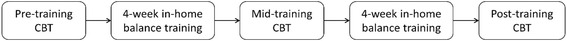
Study protocol includes three clinical balance testing (CBT) sessions and eight weeks of in-home balance training

The experimental protocol, as shown in Fig. 1, comprised pre-training assessment with clinical balance testing (CBT), eight-week in-home balance training, mid-training assessment with CBT after a four-week training period, and post-training assessment with CBT. In-home balance training started within a week of the pre-training assessment and the post-assessment was completed within one week after training.

CBT, which included eight clinical outcome measures to evaluate balance and gait performance, was completed in the clinical setting by a physical therapist blinded to the participants’ study group assignment (vibrotactile SA was not provided during CBT):

1. Activity-specific Balance Confidence (ABC, out of 100) [52]: Identifies an individual’s subjective measure of confidence in performing balance related activities of daily living. An ABC score of less than 67 indicates an increased risk for falling [53].
2. Computerized Dynamic Posturography: Sensory Organization Test (SOT) protocol [54]: Assesses an individual’s ability to use their somatosensory, visual, and vestibular systems to maintain postural stability during standing, measured by the SOT composite score. Somatosensory, visual and vestibular reliance are calculated based on ratios of SOT scores to evaluate the reliance on each sensory system as shown below.

1 $$ Somatosensory Reliance=\frac{SOTcondition2}{SOTcondition1} $$

2 $$ Visual Reliance=\frac{SOT\  condition\ 4}{SOT\  condition\ 1} $$

3 $$ Vestibular Reliance=\frac{SOT\  condition\ 5}{SOT\  condition\ 1} $$

3) Mini Balance Evaluations Systems Test (Mini-BESTest) [55]: Uses 14 items to capture anticipatory postural adjustments, reactive postural control, sensory orientation, and dynamic gait performance. The Mini-BESTest was measured with two scoring systems: total score of 28 points (MiniBESTest28) uses the lower score of the left and right sides for unilateral balance tasks; total score of 32 points (MiniBESTest32) uses the cumulative score of both sides.
4) Five Times Sit to Stand Test (5xSST) [56]: Tests functional lower limb muscle strength during transitional movements, measured in seconds. In older adults, a 5xSST duration equal to or greater than 12 s indicates a need for additional fall assessment [57].
5) Four Square Step Test (FSST) [58]: Assesses the ability to step over objects forward, sideways, and backwards, measured in seconds. FSST duration greater than 15 s for older adults indicates an increased risk for multiple falls [58].
6) Functional Reach Test (FRT) [59]: Assesses stability during maximum forward arm reach tasks while feet are in a fixed position, measured in centimeters. A FRT of less than 18 cm indicates limited mobility skills for older adults [60].
7) Ten-meter Walk Test: Assesses normal gait speed and fast gait speed, measured in meters per second. A substantial meaningful change in normal gait speed is 0.13 m/s for older adults [61].
8) Timed Up and Go (TUG) and Timed Up and Go with Cognitive Task (TUG-COG) [62]: Assesses mobility, balance, and fall risk with and without a cognitive dual-task (count backwards by three), measured in seconds. For older adults, a TUG score greater than 13.5 s or a TUG-COG score greater than 15 s indicates fall risk [63].

After CBT but prior to beginning training, the treating physical therapist (different from the blinded assessor) and study team made one initial home visit to teach the participants how to use the smart phone balance trainer (detailed in the next section) and how to correctly perform independent in-home balance training exercises. Participants performed exercises from five categories as shown in Table 1. For exercises in Categories 1 (static standing) and 2 (compliant surface standing), participants performed static balance exercises on firm and foam surfaces, respectively. For Category 3 (weight shifting) exercises, participants were instructed to shift their body to and maintain their body at a target angle for five seconds in four directions (i.e., forward, backward, left and right). Movement angle was measured on the trunk, and the target angle was determined by the research team’s physical therapists and was the same for all participants. For Category 4 (modified center of gravity) exercises, participants repeatedly raised and lowered their arms from a resting position along the sides of their bodies with palms pronated and elbows locked, to 90° of shoulder flexion. For Category 5 (gait) exercises, participants performed various overground locomotor tasks.

**Table 1:** Exercise pool modified from a recently published conceptual progression framework [64]

Category	Variables
1. Static standing^a^	Eyes, stance, head movement (yaw and pitch), cognitive tasks
2. Compliant surface standing^a^	Eyes, stance, head movement
3. Weight shifting^a^	Shifting limit, shifting speed, shifting direction
4. Modified center of gravity^a^	Arm raising speed, surface (firm, compliant and ramps), head movement
5. Gait	Walk with different speed and head movements, high march^a^, step over shoe box^a^, sidestepping, walk on heels/toes^a^, backward walking^a^, figure-of-8 walk, tandem^a^; cognitive tasks

^a^indicates the exercises for which vibrotactile SA was provided for the EG

**Fig. 2:**
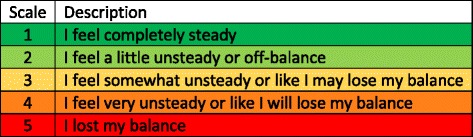
Visual analog scale used by participants to rate their stability when performing the balance exercises

Participants were asked to exercise three times per week for eight weeks (24 sessions in total). For each session, participants were given a single exercise from each of the first four categories and two exercises from the fifth category; exercises were remotely recommended by the treating physical therapist. Each exercise was performed six times for 30 s (except Category 3 exercises where the trial stopped after participants maintained the target positions for five seconds). The training duration for each session was about 45 min. Vibrotactile SA was provided to the EG via the smart phone balance trainer for all the exercises in the first four exercise categories and select exercises in the fifth category, as shown in Table 1. For these exercises, vibrotactile SA was provided during four randomly selected repetitions out of the six repetitions. The CG also wore the smart phone balance trainer, but never received vibrotactile SA. After each trial, participants were prompted by the smart phone to note any step-outs that occurred. A step-out was defined as taking a step to regain balance, touching a wall or chair for support, or opening one's eyes (on eyes closed tasks). After six repetitions participants rated their perceived stability on a visual analog scale (VAS) of 1–5 (see Fig. 2) [65]. The treating physical therapist used the reported number of step-outs and perceived stability scores to prescribe exercises weekly (three sessions per week) based on clinical experience and an exercise framework modified from a recently published conceptual progression framework [64]. The goal was to assign exercises that provided a moderate level of challenge, which was characterized by a score of 3 on the VAS. If there were no step outs and the participant rated the exercise a 1 on the VAS, a more challenging exercise was chosen; for example, adding pitch head movements would increase difficulty. If the exercises appeared too challenging (i.e., multiple step outs) and the participant rated the exercise a 4 or 5 on the VAS scale, an easier exercise was attempted until a moderately difficult exercise was found. The first set of exercises was determined during the initial home visit. Participants were asked to complete a weekly activity log to note pain that limited movement, falls, changes in medication, and any injuries from performing the exercises. MRIs were performed on a subset of the participants (*n* = 5) pre- and post-training for future analysis.

### Smart phone balance trainer

**Fig. 3:**
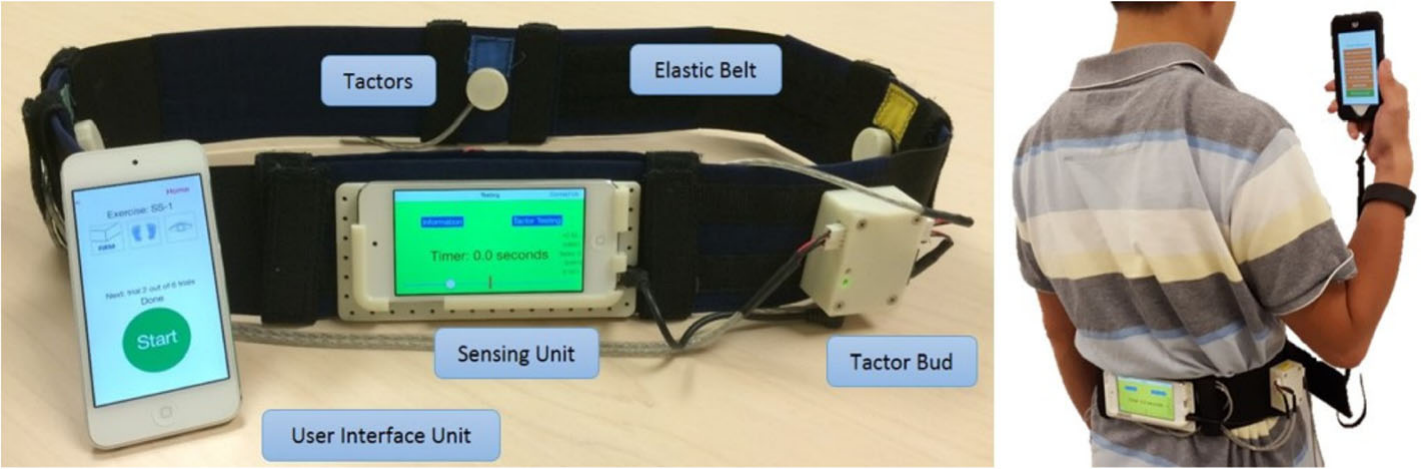
Smart phone balance trainer

A smart phone balance trainer was developed using design ethnography techniques during a co-creative design process involving engineers, physical therapists, and older adults [66]. The smart phone balance trainer comprised two Apple iPods (6th generation iPod touch, 2015), an elastic belt and a “tactor bud” accessory, as shown in Fig. 3. The two iPods are referred to as the “sensing” unit and “user interface” unit, respectively. The tactor bud contained a PCB-designed controller board, a 3.7 V battery, and four tactors (Precision Microdrives™, 310–101 vibration motors encased in plastic housings [51]). The sensing unit was attached to an elastic belt and was worn around the torso at the L4/L5 level to measure trunk sway, and the user interface unit attached to a lanyard and was worn around the neck. The four tactors were aligned over the navel, lumbar spine, and right and left sides of the torso to provide directional vibrotactile cues.

**Fig. 4:**
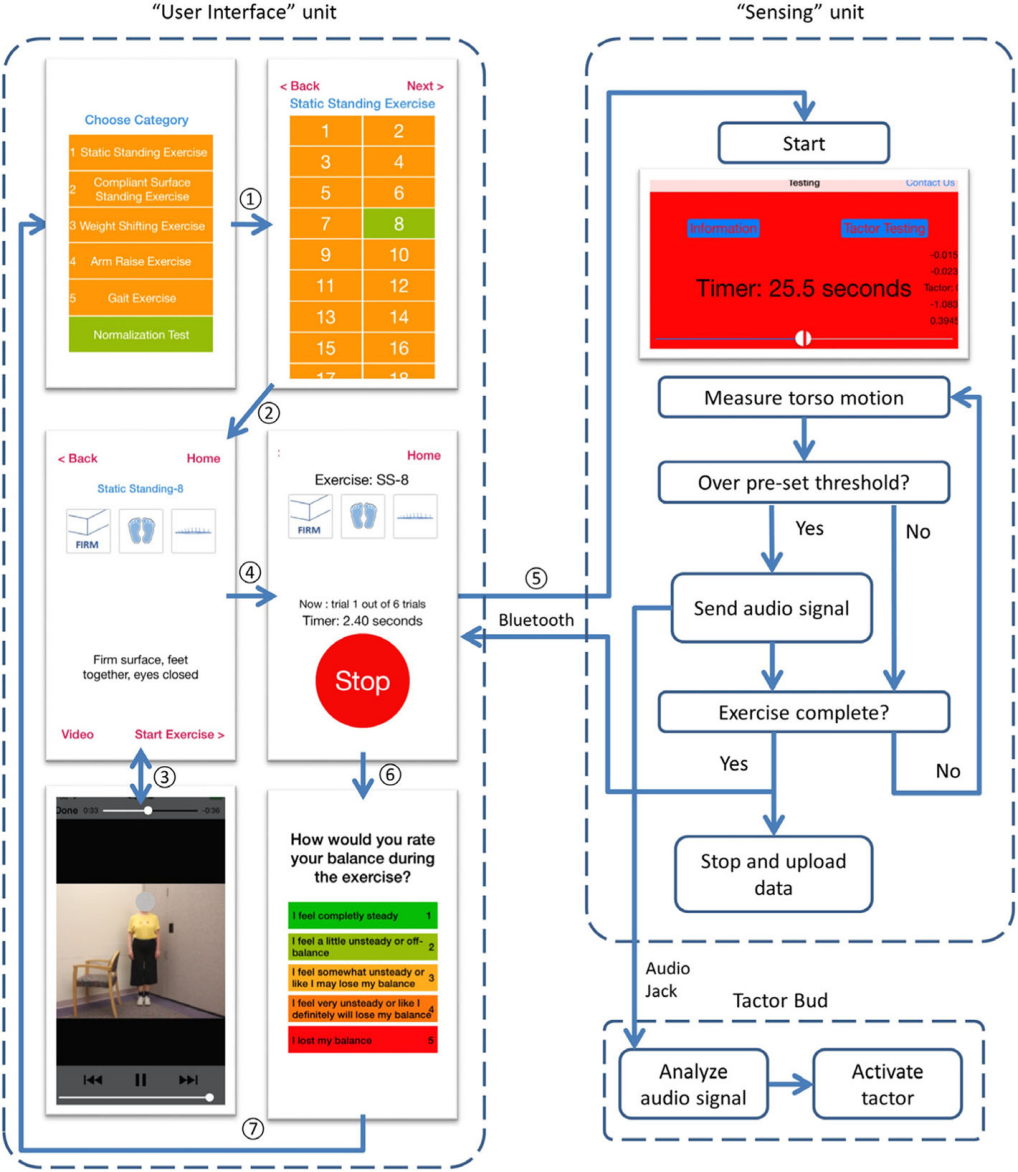
Software schematics for the user interface and sensing units

Custom software (iOS application, Apple SDK) was developed to provide a semi-automated exercise progression routine with five exercise categories for in-home training, as shown in Fig. 4. Upon the launch of the software in the user interface unit, participants were asked to select an exercise to perform. Written, graphic and video instructions were presented on screen once the exercise was selected.

During each repetition, the sensing unit used gravitational outputs (*Class CoreMotion*, Apple Inc.) to estimate angular displacements (tilt angles) in the anterior-posterior and medial-lateral directions, adopted from Lee et al.’s algorithm [51]. Angular velocities were measured by the gyroscopes. Both accelerometers and gyroscopes were sampled at 50 Hz. The user interface unit triggered the sensing unit to record trunk motion and the sensing unit informed the user interface unit of repetition completion via Bluetooth. The tactor activation signal was defined as the tilt angle plus one half times the tilt angular rate for Categories 1, 2, 4 and 5, and as the tilt angle for Category 3 exercises [24]. If the tactor activation signal exceeded a pre-set threshold [23, 38], the sensing unit sent audio output signals to the tactor bud accessory. The tactor bud accessory analyzed these audio signals and activated the corresponding tactor to provide vibrotactile cues. At the end of each repetition, the trunk motion data, number of step-outs, and visual analog scale ratings were automatically uploaded to a secured server via Wi-Fi.

### Statistical analysis

Data are presented as group mean values plus or minus (±) the standard deviation. Differences between the two groups at the pre-training assessment were tested using an independent samples two-tailed student’s t-test. The effects of training with versus without SA on the clinical outcome measures were analyzed using a linear mixed model with group (experimental, control), time (pre-, mid-, post-training) and their interaction as fixed effects and the differences among individual participants as random effects. The measurements were logarithmically transformed if they were not normally distributed (e.g., 5xSST duration, fast gait speed, and TUG-COG duration). To investigate the time effects within each group, two-tailed paired samples t-tests within each group were performed to detect statistically significant improvement, comparing mid- and post-training assessment with pre-training assessment. The significance level was set at 0.05. Bonferroni corrections were used for the paired t-tests. Due to the relatively small sample size, the minimal detectable change (MDC) was also evaluated within groups. The MDC is defined as “a statistical estimate of the smallest amount of change that can be detected by a measure that corresponds to a noticeable change in ability” [67]. It reflects the minimal amount of change in a participant’s score that ensures the change is not the result of measurement error, but is due to rehabilitation.

## Results

There were no significant differences in age or gender between the EG and CG. All participants completed the training and the three CBTs without complaints, pains, falls, or injuries, which demonstrates the feasibility of the smart phone balance trainer for in-home balance training applications.

**Fig. 5:**
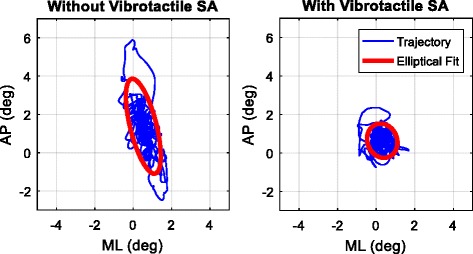
Bird’s-eye view of the body tilt trajectory in the anterior-posterior (AP) and medio-lateral (ML) directions for a sample exercise (tandem Romberg stance on firm surface with eyes open) performed by Participant 6 with and without vibrotactile SA

The data collected by the sensing unit indicated that without supervision from the study team, the EG participants were able to successfully use the vibrotactile SA in their homes to reduce their trunk sway. Fig. 5 shows illustrative data from Participant 6 performing an exercise (tandem Romberg stance on a firm surface with eyes open) in his home with and without vibrotactile SA provided by the smart phone balance trainer.

The two-tailed, independent samples t-test showed no significant differences for all clinical outcome measures between the two groups during the pre-training CBT (*p* > 0.1). Table 2 lists the results for a subset of the clinical outcome measures (SOT, MiniBESTest28, 5xSST) for all participants.

**Table 2:** Participants’ demographic information and results of a subset of clinical outcomes measures

Participant ID	Group	Age	Gender	SOT			Mini-BESTest28			5xSST duration (s)		
				Pre	Mid	Post	Pre	Mid	Post	Pre	Mid	Post
1	CG	83	M	71	81	81	21	22	25	11.0	12.5	14.6
2	EG	83	F	63	72	83	22	24	25	12.0	10.3	11.0
3	EG	70	F	78	81	76	20	26	27	10.1	9.7	8.7
4	CG	72	F	49	45	46	25	25	24	7.4	9.3	11.0
5	CG	70	M	76	79	73	26	27	22	9.7	8.4	11.4
6	EG	80	M	68	72	86	23	24	23	14.7	13.6	12.3
7	CG	73	M	60	68	65	24	25	25	8.8	10.0	8.3
8	EG	70	F	83	86	85	24	28	27	12.2	8.4	6.6
9	EG	82	F	74	78	79	25	25	26	17.8	14.2	15.0
10	CG	78	F	83	84	85	23	22	25	13.7	9.7	9.9
11	CG	74	F	68	77	76	24	24	24	17.7	9.7	9.5
12	EG	74	F	50	73	79	19	26	26	14.6	11.1	10.6

Table 3 shows the CBT assessment results for each group on average and the changes in clinical outcome measures from both pre-training to mid-training and pre-training to post-training. The linear mixed model showed significant main effects from pre-training assessments to post-training assessments in SOT composite score (*p* < 0.001), vestibular reliance (*p* < 0.01), Mini-BESTest28 (p < 0.01) and Mini-BESTest32 (*p* < 0.01) and TUG-COG duration (*p* < 0.05). The linear mixed model also showed significant interaction effects between groups from pre-training assessments to post-training assessments in SOT composite score (*p* < 0.05), Mini-BESTest28 (*p* < 0.05), and Mini-BESTest32 (*p* < 0.05). These significant interaction effects indicate greater improvements for the EG than CG with average increases of 1.1, 0.40, and 0.58 points per week for the SOT composite scores, Mini-BESTest28, and Mini-BESTest32, respectively. There were no significant interaction effects for the other CBT outcomes. The within-group paired t-test showed significant improvements for the EG in 5xSST duration during both mid- (*p* < 0.01) and post-training (*p* < 0.01). For the CG, there were no significant improvements on any of the CBT outcomes.

**Table 3:** Clinical outcome measure results for pre-, mid- and post-training CBT and changes from pre-training CBT for the EG and CG. Average values with standard deviations are shown

	Experimental Group					Control Group				
	Pre	Mid	Post	Mid - Pre	Post - Pre	Pre	Mid	Post	Mid - Pre	Post - Pre
ABC score	90.9 ± 3.5	89.4 ± 6.4	91.3 ± 6.3	−1.5 ± 8.1	0.5 ± 8.7	94.0 ± 3.5	91.8 ± 4.2	93.8 ± 3.2	−2.2 ± 2.8	−0.2 ± 2.4
SOT score^a, b^	69.3 ± 11.8	77.0 ± 5.7	81.3 ± 3.9	7.7 ± 7.8	12.0 ± 12.1	67.8 ± 5.1	72.3 ± 12.0	71.0 ± 14.5	4.5 ± 5.5	3.2 ± 5.5
Somatosensory reliance	0.98 ± 0.02	0.98 ± 0.02	0.98 ± 0.02	0.00 ± 0.03	0.01 ± 0.04	0.95 ± 0.02	0.96 ± 0.02	0.95 ± 0.02	0.01 ± 0.03	0.00 ± 0.02
Visual reliance	0.82 ± 0.11	0.86 ± 0.07	0.89 ± 0.03	0.04 ± 0.11	0.07 ± 0.11	0.87 ± 0.05	0.87 ± 0.09	0.88 ± 0.10	0.01 ± 0.06	0.01 ± 0.08
Vestibular reliance^a^	0.53 ± 0.21	0.63 ± 0.20	0.74 ± 0.06	0.10 ± 0.08	0.21 ± 0.17	0.40 ± 0.28	0.57 ± 0.33	0.56 ± 0.31	0.17 ± 0.23	0.16 ± 0.24
Mini-BESTest28^a,b^	22.2 ± 2.3	25.5 ± 1.5	25.7 ± 1.5	3.3 ± 2.8	3.5 ± 2.9	23.8 ± 1.7	24.2 ± 1.9	24.2 ± 1.2	0.3 ± 0.8	0.3 ± 2.7
Mini-BESTest32^a,b^	25.0 ± 2.6	28.7 ± 2.2	29.3 ± 1.9	3.7 ± 3.5	4.3 ± 3.4	26.8 ± 2.0	27.5 ± 2.7	26.5 ± 1.4	0.7 ± 1.6	−0.3 ± 3.2
5xSST duration (s)	13.5 ± 2.7	11.2 ± 2.3	10.7 ± 2.9	−2.4 ± 1.4^c^	−2.9 ± 1.7^c^	11.4 ± 3.8	9.9 ± 1.4	10.8 ± 2.2	−1.5 ± 3.9	−0.6 ± 4.7
FSST duration (s)	9.9 ± 2.2	9.4 ± 2.3	9.5 ± 2.1	−0.6 ± 0.6	−0.6 ± 0.5	10.5 ± 1.8	10.8 ± 1.5	10.1 ± 2.2	0.3 ± 1.5	−0.4 ± 1.2
FRT (cm)	35.0 ± 6.1	29.9 ± 5.75	31.9 ± 5.0	−5.1 ± 4.3	−3.0 ± 4.2	31.9 ± 5.2	28.9 ± 3.3	35.0 ± 2.7	−3.0 ± 4.1	3.1 ± 3.8
Normal gait speed (m/s)	1.22 ± 0.08	1.28 ± 0.16	1.27 ± 0.19	0.06 ± 0.17	0.06 ± 0.20	1.29 ± 0.11	1.23 ± 0.14	1.26 ± 0.16	−0.07 ± 0.07	−0.04 ± 0.15
Fast gait speed (m/s)	1.62 ± 0.24	1.70 ± 0.28	1.69 ± 0.29	0.07 ± 0.21	0.06 ± 0.31	1.58 ± 0.11	1.57 ± 0.18	1.54 ± 0.19	−0.01 ± 0.10	−0.04 ± 0.16
TUG duration (s)	10.8 ± 2.1	9.6 ± 1.2	10.5 ± 1.4	−1.1 ± 1.8	−0.3 ± 1.4	10.0 ± 1.3	9.8 ± 1.6	9.8 ± 1.1	−0.2 ± 1.0	−0.2 ± 1.6
TUG-COG duration (s)^a^	13.3 ± 2.9	11.6 ± 1.6	11.8 ± 2.1	−1.7 ± 1.8	−1.5 ± 1.4	10.9 ± 2.7	11.0 ± 2.3	9.7 ± 1.6	0.2 ± 1.8	−1.1 ± 2.8

Superscripts indicate ^a^significant main effects, ^b^significant interaction effects from the linear mixed model (*p* < 0.05), and ^c^significant differences from the group-paired t-tests (*p* < 0.017)

## Discussion

This is the first study to investigate the effects of long-term (eight-week) balance training with and without vibrotactile SA on clinical outcome measures for community-dwelling older adults. Analysis of the twelve participants’ scores showed that both the EG and CG had significant improvements in SOT composite scores, vestibular reliance, Mini-BESTest28, Mini-BESTest32 and TUG-COG duration; however, the EG improved significantly more than the CG in SOT composite scores, Mini-BESTest28, and Mini-BESTest32. In addition, significant improvements in 5xSST duration were found within the EG, whereas no significant improvements were found within the CG. However, no significant improvements were found in the ABC score, somatosensory reliance, visual reliance, FSST duration, FRT, gait speed, and TUG duration.

After training, both groups showed improvements in the SOT composite score; significantly greater improvements were found for participants trained with SA than without SA (8 points vs. 5 points at mid-training, 12 points vs. 3 points at post-training on average). Prior studies have shown that when SA was provided, real-time sway reductions were noted for exercises in the SOT protocol [68]. The results of the current study indicate that after long-term training with SA, balance improvements in SOT protocol can be retained even when SA was not provided. SOT composite score improvements after training with SA (2–8 weeks) have been demonstrated in people with Parkinson’s disease (~18 points) [43], people with bilateral vestibular disorders (~9 points) [45, 69], and people with other balance disorders (~8 points) [44]. Although the participants in our study reported no specific balance disorders, the EG exhibited similar improvements in SOT composite score (~12 points) after long-term training with SA. The MDC for the SOT composite score for young adults was previously determined to be greater than 8.1 points [70]. In this study, three participants in the EG achieved MDCs in SOT composite scores, while only one participant in the CG achieved a MDC. Furthermore, these three EG participants improved by at least 15 points, while the CG participant improved by 10 points. These results indicate that training with SA may be more effective than training alone for achieving MDCs in SOT performance. From mid-training to post-training, the EG showed continuous improvements, while the CG showed a plateau effect, which suggests that training with SA could result in higher potential improvement than training without SA.

Somatosensory, visual, and vestibular reliance were calculated using SOT Conditions 1, 2, 4, and 5. Somatosensory reliance did not significantly improve following training, however, the margin for improvement was limited by high levels of somatosensory reliance prior to training. Participants in both the EG and CG relied more on visual and vestibular inputs for maintaining balance after training, although vestibular reliance showed a larger increase. These shifts in visual and vestibular reliance may support the “reweighting” hypothesis for balance training [41]. Increased vestibular reliance may be attributed to performing exercises with eyes closed and/or incorporating head movements [71]. Moreover, greater increases on vestibular reliance were observed in the EG, which may suggest that training with SA has a greater impact on reweighting vestibular inputs than training without SA.

The EG showed significantly greater improvements than the CG for both Mini-BESTest28 and Mini-BESTest32 scores. Training with SA while performing static and dynamic standing and gait exercises could explain this difference because the Mini-BESTest assesses dynamic balance [55]. Although no MDC data are available for older adults, Godi et al. reported a 3.5-point MDC for the Mini-BESTest28 among people with Parkinson’s disease (baseline Mini-BESTest28 value was 12.8 points) [72]. In our study, three participants in the EG demonstrated a 3.5-point change during mid-training CBT versus no participants in the CG. The average baseline Mini-BESTest28 value for all participants was 22.2 points.

Within-group analysis of 5xSST performance showed significant improvement of test duration for the EG but not for the CG after training concluded. Additionally, all participants in the EG improved their 5xSST durations after training, but only three out of the six participants in the CG showed improvements. Given that improvements in 5xSST duration are correlated with improved lower limb muscle strength and stability during transitional movements [56], training with SA may be more effective for improving functional mobility. It was also noted that two participants from each group reduced their 5xSST durations to less than 12 s (a fall risk indicator [57]) at the mid- and post-training CBTs. Finally, although the sit-to-stand task was not an exercise performed during balance training, training effects from dynamic standing tasks (especially Category 3, Weight Shifting exercises, and Category 4, Modified Center of Gravity exercises) may have been transferred to the sit-to-stand task.

No significant changes in ABC scores were found in either the EG or the CG, although scores generally declined from pre-training to mid-training and increased from mid-training to post-training. As healthy older adults, all participants had relatively high ABC scores (>85) at the pre-training assessment, and were therefore unlikely to show further improvement in balance confidence (i.e., ceiling effect) [73]. Declines in scores from pre- to mid-training are consistent with an initial overconfidence in balance abilities and a shift in awareness of limitations [74]. Score increases from mid- to post-training may reflect improvements in overall balance performance, although the improvements were relatively small.

The EG and CG showed significant improvements in TUG-COG duration, possibly due to the effects of training with cognitive tasks. However, no significant improvements for FRT distance, FSST duration, normal gait speed, fast gait speed, and TUG duration were noted. The lack of significant improvements may have been due to ceiling effects, or the difficulty level of the selected exercises may have been below the necessary level to elicit improvements for these outcomes. However, on average, the EG showed greater improvements in normal gait speed and fast gait speed than the CG. Additionally, one participant in the EG achieved a MDC for normal gait speed (0.18 m/s for Parkinson’s Disease) and two participants in the EG achieved a MDC for fast gait speed (0.25 m/s for Parkinson’s Disease) [75]. No participants achieved MDCs in the CG. Transfer effects from using SA during static and dynamic exercises may account for observed differences.

Our findings appear to contradict those of Lim et al., who found no significant differences in body sway between a group training with SA and a group training without SA after a two-week program [50]; however, these two studies have several important differences. First, participants in Lim et al.’s study trained for two weeks (3×/week) with six training sessions in total, whereas in this study all participants trained for eight weeks (3×/week) with 24 training sessions in total. As shown by Lesinski et al., longer training periods result in greater improvements in balance performance [16]. Second, Lim et al.’s study provided SA to the experimental group during all exercise repetitions during all sessions (i.e., 100% feedback), whereas this study provided SA to the EG for four out of the six repetitions for each exercise (i.e., 67% feedback). Feedback can have negative effects if provided too frequently. Winstein and Schmidt found that providing feedback for the entire duration of motor skill training can improve short term performance but limit motor learning, while providing feedback for only portions of training produces poor initial performance results but improves motor skill retention [76, 77]. Therefore, training with reduced feedback frequency in this study may have improved skill retention after training concluded. Third, Lim et al. trained all participants using the same exercises throughout all sessions regardless of the participants' balance abilities, whereas in this study a physical therapist selected the exercises performed by each participant based on his/her historical performance. Training with a constant set of exercises may limit the margin for improvement among high functioning participants, while those with poorer balance ability may experience larger improvements. In balance rehabilitation programs, experienced physical therapists progress balance exercises to achieve greater balance improvements [64]. Individualized exercise selection in this study allows participants to perform progressively challenging exercises throughout the entire training program to maximize improvement regardless of skill level. Fourth, Lim et al. quantified the effects of balance training by comparing measures of trunk motion, whereas this study used clinical outcome measures to evaluate improvements.

This study employed some elements of telerehabilitation to monitor performance and provide custom exercise regimens. Body motion, subjective ratings of balance and number of step outs were captured on the smart phone balance trainer during home-based balance training. This information was sent to a physical therapist via a wireless internet connection. Exercise programs were customized based on performance data and updated regimens were sent from the physical therapist to the participants via email. Conceptually, this aligns with telerehabilitation models, which deliver remote rehabilitation services, including assessments and interventions, via telecommunication networks [78]. However, our paradigm required less expert (i.e., physical therapist) engagement with participants as compared to traditional programs because we provided within-session vibrotactile SA as instructional balance cues. Physical therapist’s time commitments were limited to less than thirty minutes per week per participant and focused on analysis of previous balance performance and customization of the rehabilitation program, whereas typical telerehabilitation programs generally engage the expert and user remotely for the duration of the training session [79]. The findings of this study are consistent with prior work that has shown improved balance performance following telerehabilitation interventions for both people with balance deficits and community dwelling older adults [79–83]. Previously published research has also demonstrated potential economic benefits to using telerehabilitation approaches [79, 80]. While cost effectiveness was not explored as part of this study, the smart phone balance trainer (<$1 k) coupled with reduced patient-expert interaction could reduce the overall cost of providing rehabilitative care for a subset of people with balance deficits and simultaneously mitigate future costs stemming from injurious loss-of-balance events. Overall, improvements in clinical outcomes support the potential use of a smart phone balance trainer as a telerehabilitation tool.

Our study is not without limitations. First, vibrotactile SA was only provided during a subset of exercises under the gait category because few studies have addressed the effectiveness of SA for improving stability during locomotor tasks [22]. Typical feedback strategies during gait activities include walking in step with auditory or visual cues or vibrotactile cues presented to a single body segment or joint to warn of extension beyond a desired angle [32, 38, 84]. Sienko et al. provided continuous vibrotactile SA based on trunk motion during overground locomotion, but slightly reduced trunk sway was observed in a subset of the trials and some participants demonstrated stiffening in the coronal plane [38]. Second, although this study employed an experienced physical therapist to instruct participants on correct exercise performance, provided handouts with instructional text and pictures, and provided exercise videos, correctness of exercise performance was not monitored during training because training occurred in participants’ homes. Third, despite the statistical and clinical significances found in this study, the sample size was relatively small. Finally, although this study used an experienced physical therapist to recommend the exercises remotely, the information provided to the physical therapist by the smart phone balance trainer was limited to the number of step-outs in the six repetitions and the stability perception ratings from the participants. A more sophisticated algorithm that captures exercise performance more comprehensively could help therapists make better recommendations in the future.

## Conclusions

In-home balance training with vibrotactile SA for eight weeks improved balance performance of community-dwelling healthy older adults in this preliminary study. Participants trained with SA improved more than those trained without SA, particularly in SOT composite, Mini-BESTest, and 5xSST performance. Balance training with SA also increased visual and vestibular reliance, and improved static and dynamic balance, compared to training without SA. The lack of significant improvements in gait-related clinical outcome measures may be due to the lack of meaningful SA provided when performing gait exercises and the limited transfer effects from performance of standing exercises. All participants completed the eight-week training protocol with no reports of pain, injuries, or falls, which suggests that healthy older adults are able to use the smart phone balance trainer safely and independently. Overall, this study supports SA as a balance rehabilitation tool and potential telerehabilitation tool for use by community-dwelling healthy older adults.

## Abbreviations

5xSST: Five Times Sit to Stand Test
ABC: Activity-specific Balance Confidence
CBT: Clinical Balance Test
CG: Control Group
EG: Experimental Group
FRT: Functional Reach Test
FSST: Four Square Step Test
MDC: Minimal Detectable Change
Mini-BESTest: Mini Balance Evaluations Systems Test
SA: Sensory Augmentation
SOT: Sensory Organization Test
TUG: Timed Up and Go
TUG-COG: Timed Up and Go with Cognitive Task
VAS: Visual Analog Scale
